# Supplementary material for: Cross-domain metabolic interactions link Methanobrevibacter smithii to colorectal cancer microbial ecosystems
Source: Nat Commun. 2026 Feb 20;17:2979. doi: 10.1038/s41467-026-69711-7 (PMC13035836; doi:10.1038/s41467-026-69711-7)
Supplement: Supplementary file 1 — Supplementary Information [file 41467_2026_69711_MOESM1_ESM.pdf]

Supplementary information

Cross-domain metabolic interactions link *Methanobrevibacter smithii* to colorectal cancer microbial ecosystems

Rokhsareh Mohammadzadeh[1], Alexander Mahnert[1], Tamara Zurabischvili[1], Lisa Wink[1], Christina Kumpitsch[1], Hansjoerg Habisch[2], Jannik Sprengel[3,4], Klara Filek[1], Polona Mertelj[1], Dominique Pernitsch[5], Kerstin Hingerl[5], Marija Durdevic[6,7], Gregor Gorkiewicz[6], Christian Diener[1], Alexander Loy[8], Dagmar Kolb[5], Christoph Trautwein[3,4,9,10,11], Tobias Madl[2,12], Christine Moissl-Eichinger*[1,12]

[1] Diagnostic and Research Institute of Hygiene, Microbiology and Environmental Medicine, Medical University of Graz, 8010 Graz, Austria
[2] Otto Loewi Research Center, Medicinal Chemistry, Medical University of Graz, Graz, Austria
[3] Core Facility Metabolomics, Medical Faculty University of Tübingen, Tübingen, Germany
[4] M3 Research Center for Malignome, Metabolome & Microbiome, Medical Faculty University of Tübingen, Tübingen, Germany
[5] Core Facility Ultrastructure Analysis, Medical University of Graz, Graz, Austria
[6] Institute of Pathology, Medical University of Graz, Graz, Austria
[7] Core Facility Computational Bioanalytics, Center for Medical Research, Medical University of Graz, Graz, Austria
[8] Division of Microbial Ecology, Centre for Microbiology and Environmental Systems Science, University of Vienna, Vienna, Austria
[9] Department of Preclinical Imaging and Radiopharmacy, Werner Siemens Imaging Center, University Hospital Tübingen, Tübingen, Germany
[10] Cluster of Excellence CMFI (EXC 2124) "Controlling Microbes to Fight Infections", Eberhard Karls University of Tübingen, Tübingen, Germany
[11] Cluster of Excellence iFIT (EXC 2180) "Image Guided and Functionally Instructed Tumor Therapies", University of Tübingen, Tübingen, Germany
[12] BioTechMed, Graz, Austria

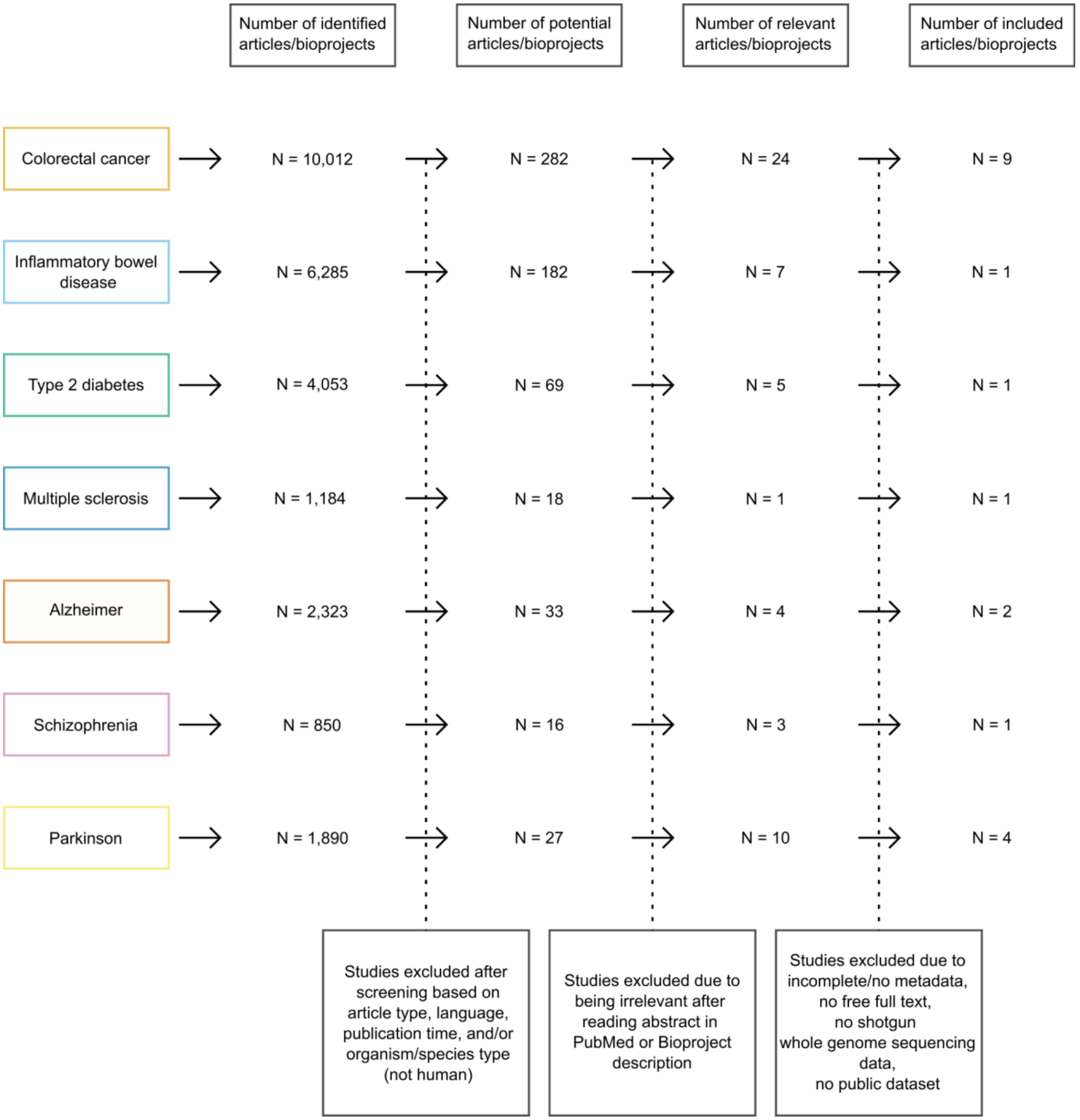

Supplementary Fig. 1. Study selection and overview of included datasets for meta-analysis. A total of 631 studies were initially screened for potential inclusion in the meta-analysis. Following eligibility assessment, 19 studies were retained, comprising 9 colorectal cancer (CRC), 1 inflammatory bowel disease (IBD), including Crohn's disease (CD) and ulcerative colitis (UC), 1 type 2 diabetes (T2D), 1 multiple sclerosis (MS), 2 pre-Alzheimer's disease (pre-AD; initially searched for Alzheimer's patients, but due to the low sample sizes, we only included those with pre-AD), 1 schizophrenia (SCZ), and 4 Parkinson's disease (PD) datasets.

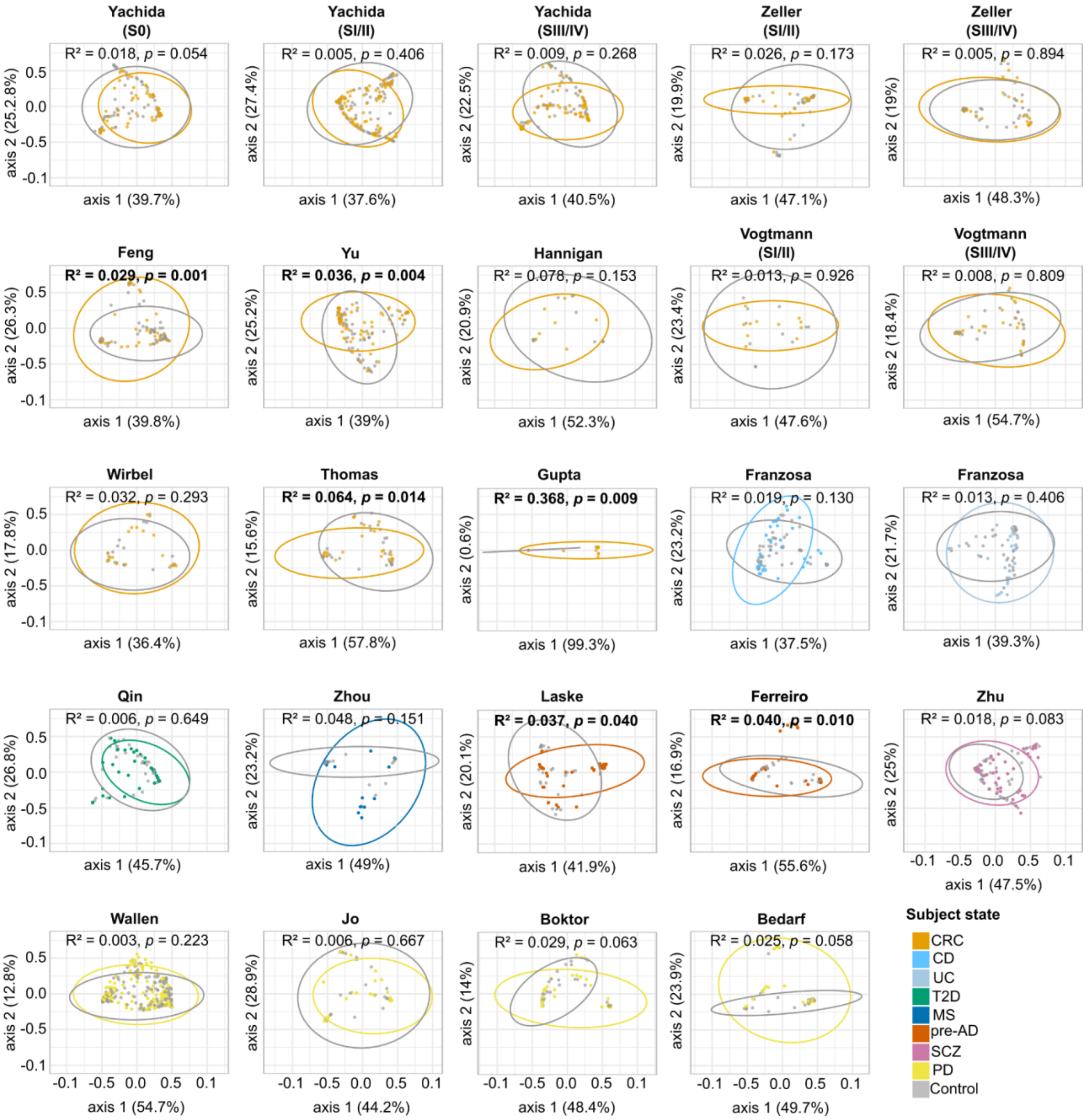

Supplementary Fig. 2. Variation in archaeal community structure across study populations, assessed by Principal Coordinate Analysis (PCoA) using Bray-Curtis distance to compare control and diseased subjects. Beta-diversity was assessed using species-level abundance profiles normalized by total sum scaling (TSS). Group variability was visualized with ellipses, and statistical differences were tested using PERMANOVA (999 permutations). Significant *p*-values (*p*-values < 0.05) are shown in bold. For the Yachida[1], Zeller[2], and Vogtmann[3] cohorts, where sufficient information and sample sizes were available, subjects were further stratified by colorectal cancer stage. CRC, Colorectal cancer; S0, Stage 0; SI/SII, Stage I/II; SIII/SIV, Stage III/IV; CD, Crohn's disease; UC, Ulcerative Colitis; T2D, Type 2 Diabetes; MS, Multiple Sclerosis; pre-AD, pre-Alzheimer's Disease; SCZ, Schizophrenia; PD, Parkinson's Disease. Source data are provided as a Source Data file.

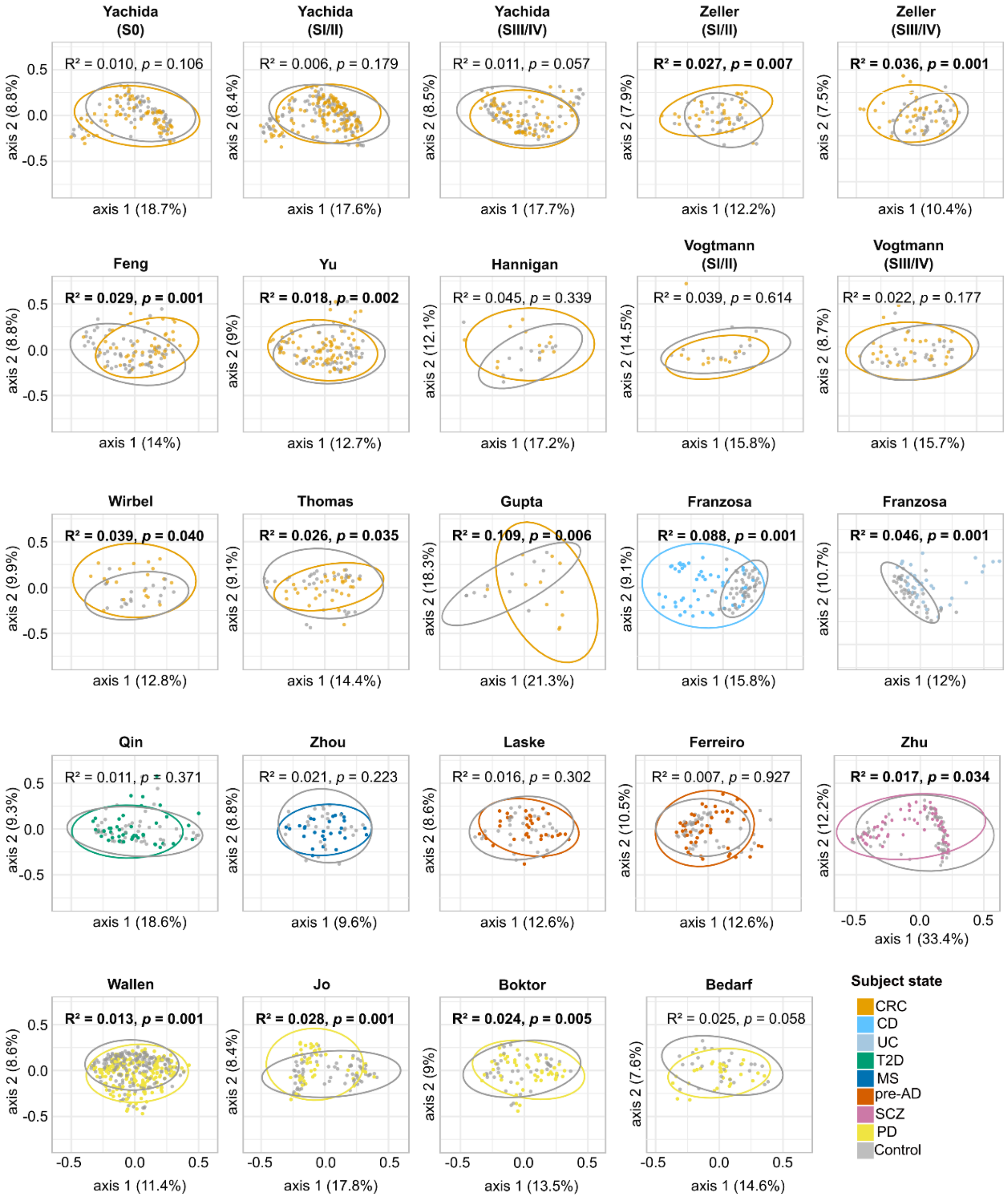

Supplementary Fig. 3. Variation in bacterial community structure across study populations, assessed by Principal Coordinate Analysis (PCoA) using Bray-Curtis distance to compare control and diseased subjects. Beta-diversity was assessed using species-level abundance profiles normalized by total sum scaling (TSS). Group variability was visualized with ellipses, and statistical differences were tested using PERMANOVA (999 permutations). Significant *p*-values (*p*-values < 0.05) are shown in bold. For the Yachida[1], Zeller[2], and Vogtmann[3] cohorts, where sufficient information and sample sizes were available, subjects were further stratified by colorectal cancer stage. CRC, Colorectal cancer; S0, Stage 0; SI/SII, Stage I/II; SIII/SIV, Stage III/IV; CD, Crohn's disease; UC, Ulcerative Colitis; T2D, Type 2 Diabetes; MS, Multiple Sclerosis; pre-AD, pre-Alzheimer's Disease; SCZ, Schizophrenia; PD, Parkinson's Disease. Source data are provided as a Source Data file.

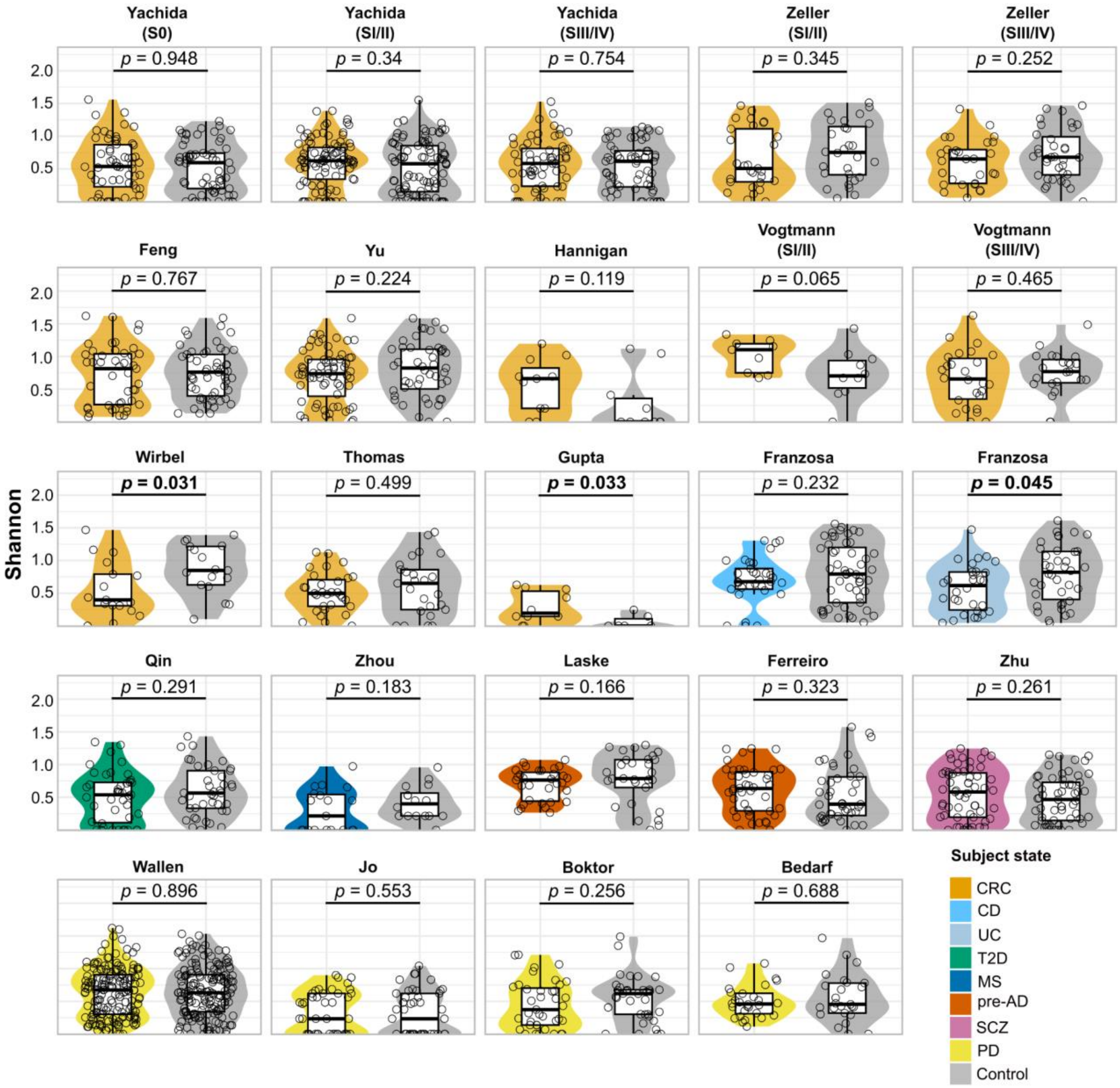

Supplementary Fig. 4. Comparison of gut archaeal diversity between disease and control groups across datasets, based on the Shannon diversity index. Case–control differences were tested with the two-sided Wilcoxon rank-sum test. Significant *p*-values (*p*-values < 0.05) are shown in bold. For the Yachida[1], Zeller[2], and Vogtmann[3] cohorts, where sufficient information and sample sizes were available, subjects were further stratified by colorectal cancer stage. CRC, Colorectal cancer; S0, Stage 0; SI/SII, Stage I/II; SIII/SIV, Stage III/IV; CD, Crohn's disease; UC, Ulcerative Colitis; T2D, Type 2 Diabetes; MS, Multiple Sclerosis; pre-AD, pre-Alzheimer's Disease; SCZ, Schizophrenia; PD, Parkinson's Disease. Source data are provided as a Source Data file.

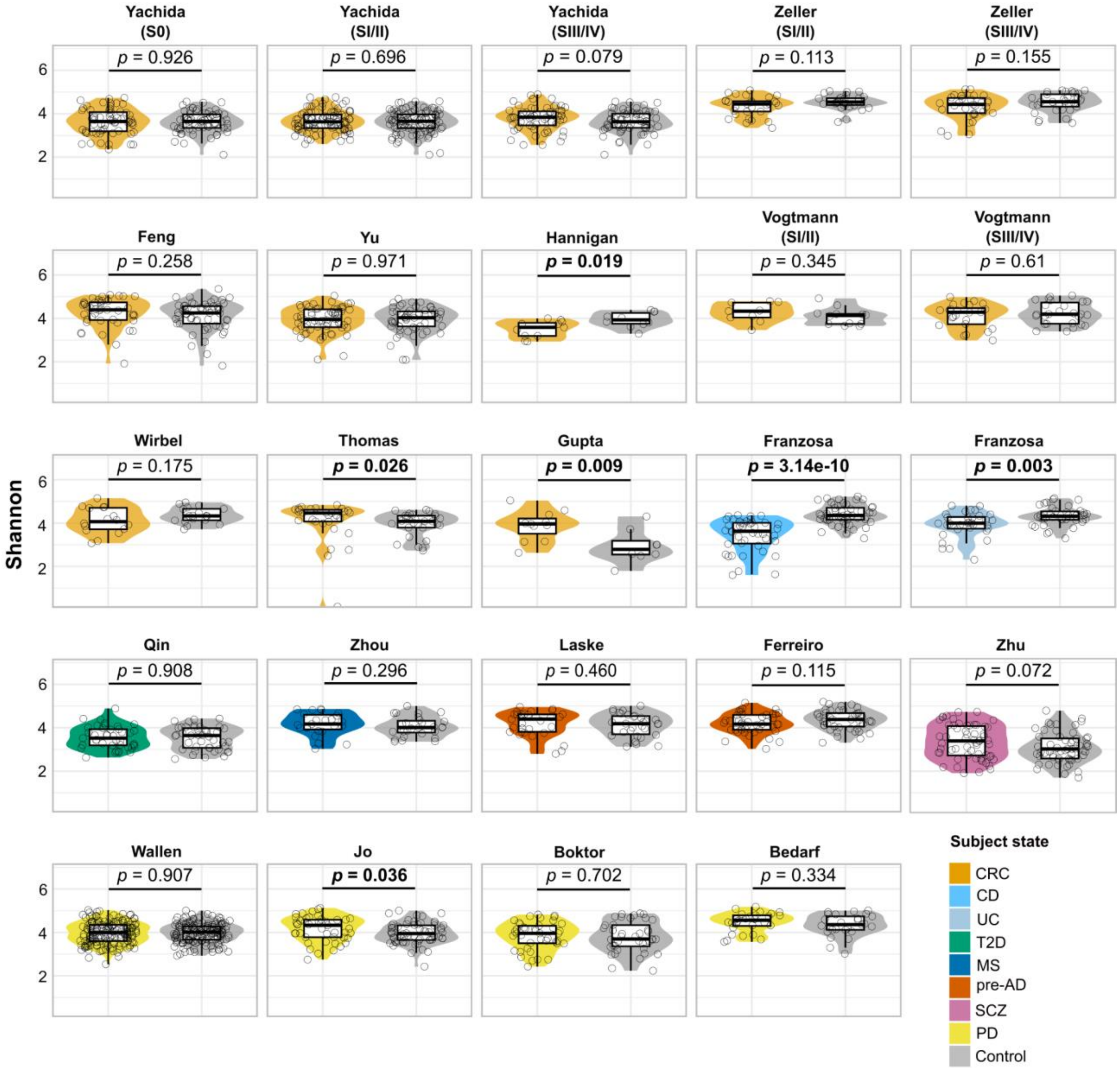

Supplementary Fig. 5. Comparison of gut bacterial diversity between disease and control groups across datasets, based on the Shannon diversity index. Case–control differences were tested with the two-sided Wilcoxon rank-sum test. Significant *p*-values (*p*-values < 0.05) are shown in bold. For the Yachida[1], Zeller[2], and Vogtmann[3] cohorts, where sufficient information and sample sizes were available, subjects were further stratified by colorectal cancer stage. CRC, Colorectal cancer; S0, Stage 0; SI/SII, Stage I/II; SIII/SIV, Stage III/IV; CD, Crohn's disease; UC, Ulcerative Colitis; T2D, Type 2 Diabetes; MS, Multiple Sclerosis; pre-AD, pre-Alzheimer's Disease; SCZ, Schizophrenia; PD, Parkinson's Disease. Source data are provided as a Source Data file.

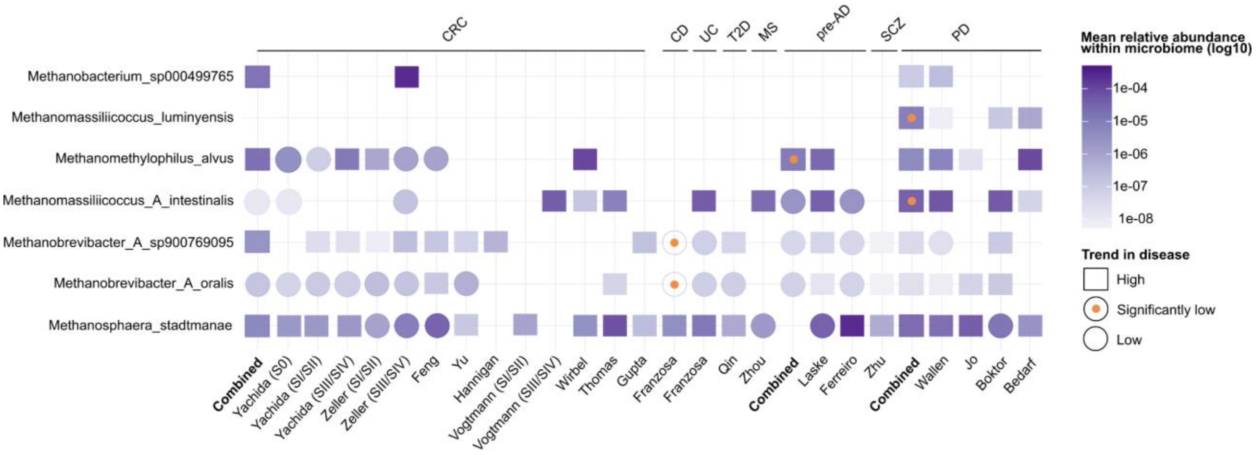

Supplementary Fig. 6. Differential abundance patterns of less dominant methanogenic archaea. differential abundance testing was performed for methanogenic archaeal species not ranked among the top five most abundant within each study. species are represented each with a symbol indicating the direction of abundance change between disease and control groups: squares denote higher abundance in disease, and circles denote lower abundance in disease. An orange dot inside the symbol indicates a statistically significant difference (p-adjusted < 0.05), while empty symbols represent non-significant trends. Differential abundance was assessed using CLR-transformed data and two-sided Wilcoxon rank-sum test with FDR correction. For the Yachida[1], Zeller[2], and Vogtmann[3] cohorts, where sufficient information and sample sizes were available, subjects were further stratified by colorectal cancer stage. CRC, Colorectal cancer; S0, Stage 0; SI/SII, Stage I/II; SIII/SIV, Stage III/IV; CD, Crohn's disease; UC, Ulcerative Colitis; T2D, Type 2 Diabetes; MS, Multiple Sclerosis; pre-AD, pre-Alzheimer's Disease; SCZ, Schizophrenia; PD, Parkinson's Disease. Source data are provided as a Source Data file.

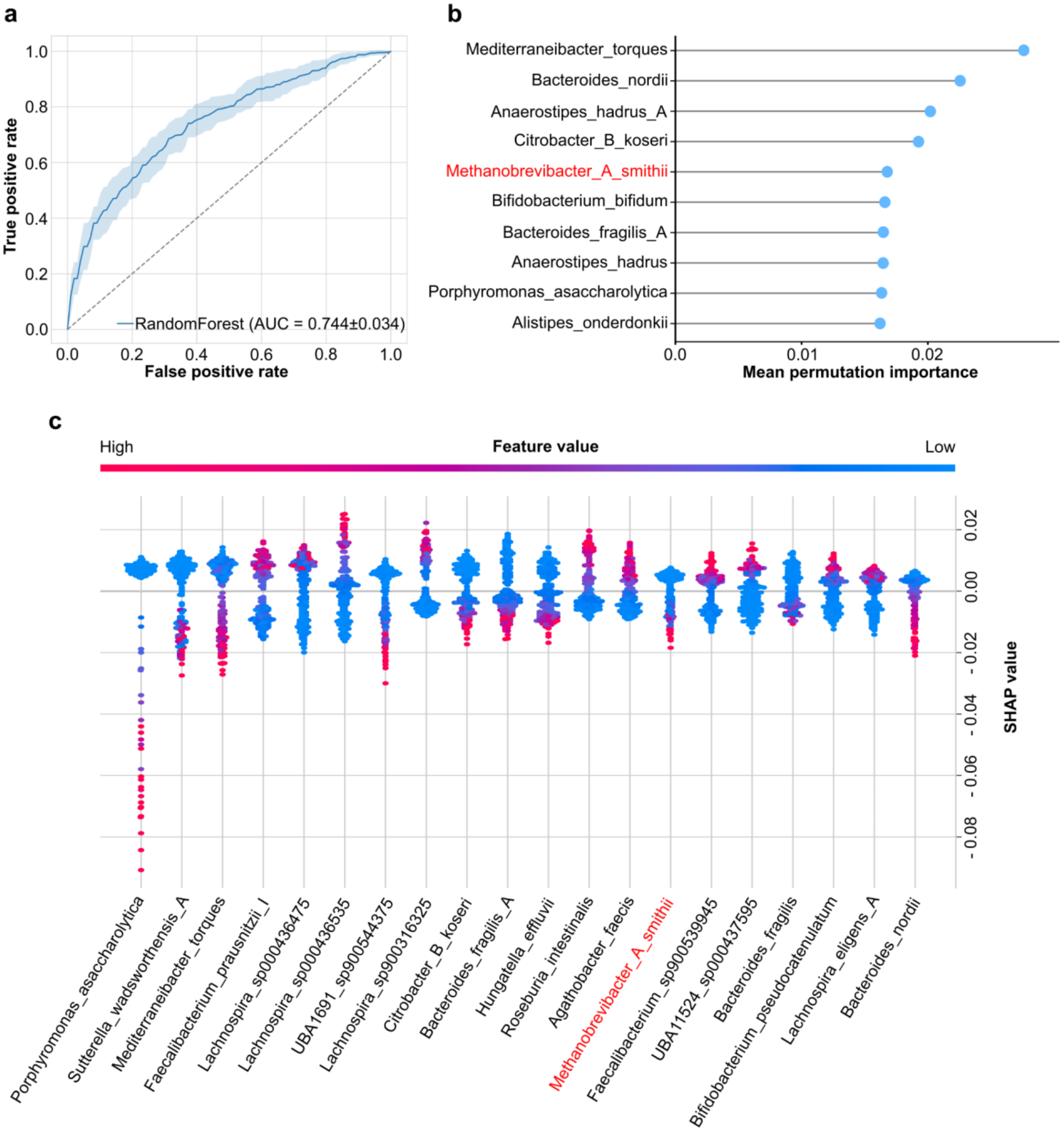

Supplementary Fig. 7. Diagnostic performance of microbial species in distinguishing colorectal cancer patients from controls. **a** AUC analysis showing the diagnostic performance of the microbial species in discriminating CRC patients from HC in the pooled dataset. **b** The importance of top 10 microbial species in the diagnostic model with pooled data with 1000 iterations on the pooled dataset, including Methanobrevibacter_A_smithii (in red). **c** SHAP summary plots depicting the relative contribution of the top 20 features driving classification between CRC and control samples. Each point represents an individual sample, with SHAP values on the x-axis quantifying the magnitude and direction of each feature's influence on the model output. Color gradients represent feature intensity (high values in red, low values in blue). Features with negative SHAP values shift predictions toward CRC, including Methanobrevibacter_A_smithii (in red); whereas positive SHAP values shift predictions toward the healthy state. Source data are provided as a Source Data file.

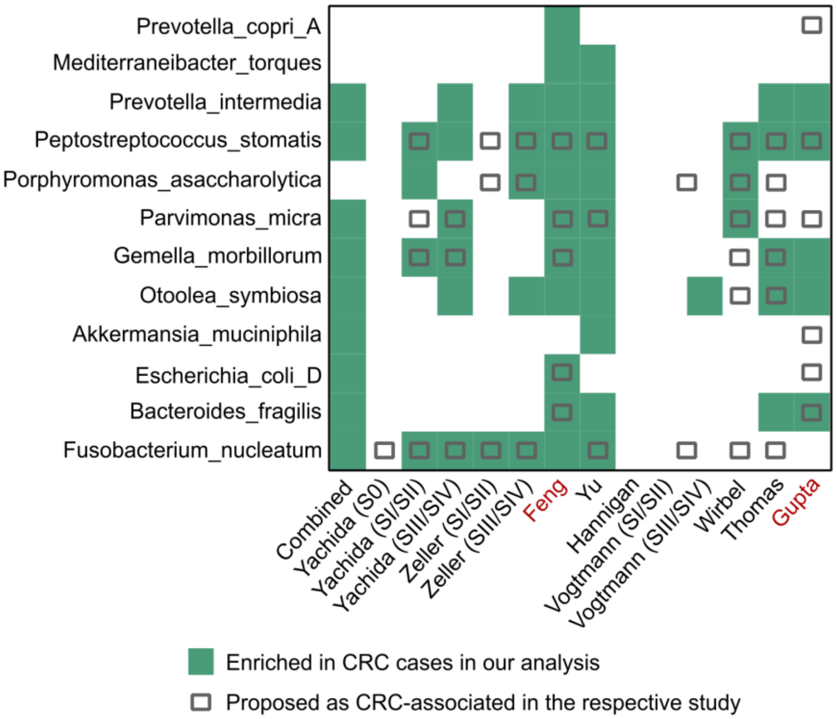

Supplementary Fig. 8. Overview of bacterial species previously reported to be associated with colorectal cancer (CRC). Green color indicates enrichment (p-adjusted < 0.05) in CRC based on our reanalysis of combined datasets, and each dataset separately after matching case-control samples for confounding variables (age, sex, and BMI). Small grey squares represent associations reported in the original publications. The study by Vogtmann et al.[3] reported *Porphyromonas* and *Fusobacterium* as enriched at the genus level only, and the differential abundance testing between CRC stages were not investigated, while Hannigan et al.[4] focused exclusively on the virome rather than the full microbiome. Studies showing significant increase of Methanobrevibacter_A_smithii are highlighted in red. Differential abundance was assessed using CLR-transformed data and two-sided Wilcoxon rank-sum test with FDR correction. S0, Stage 0; SI/SII, Stage I/II; SIII/SIV, Stage III/IV. Source data are provided as a Source Data file.

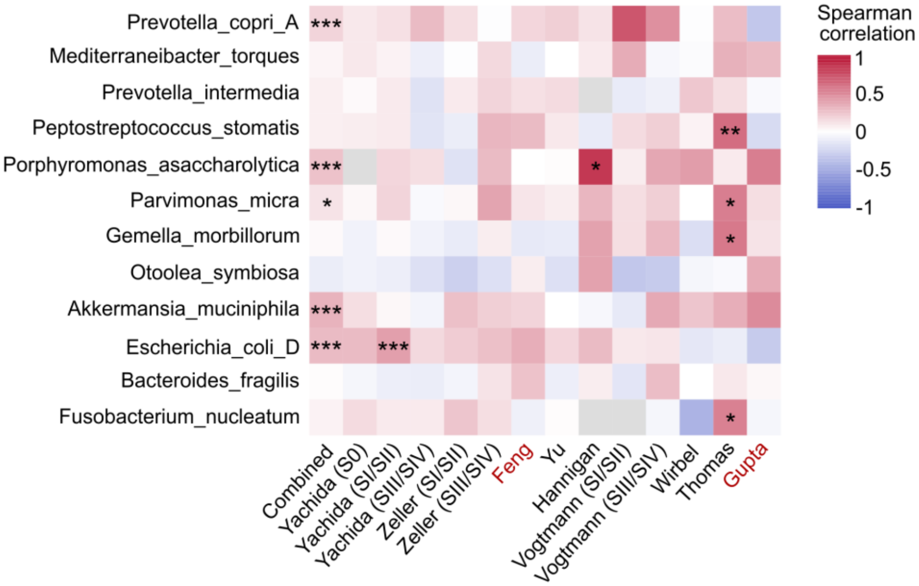

Supplementary Fig. 9. Heatmap displaying correlations between selected CRC-bacterial markers and Methanobrevibacter_A_smithii in CRC microbiome samples based on Spearman's rho correlation. Individual studies showing significant increase of Methanobrevibacter_A_smithii are highlighted in red. $p < 0.05$ (*), $p < 0.01$ (**), $p < 0.001$ (***). Source data are provided as a Source Data file.

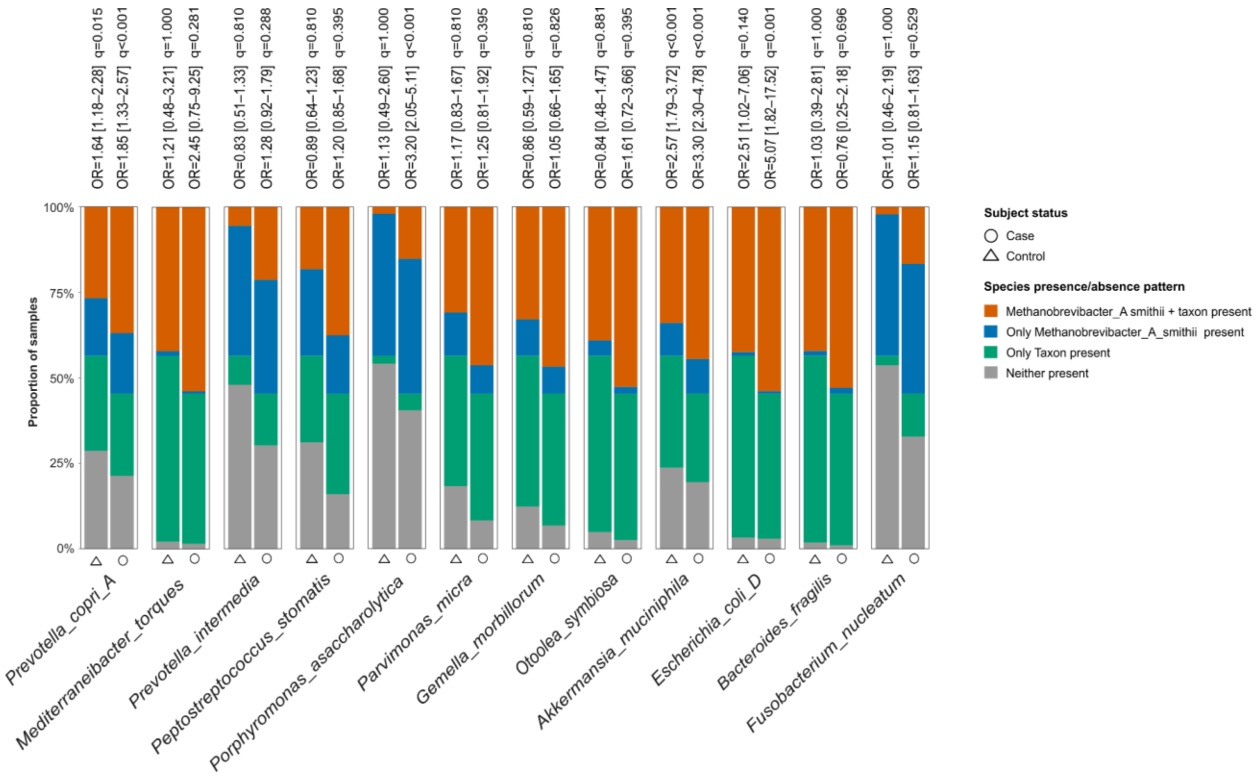

Supplementary Fig. 10. Co-occurrence of Methanobrevibacter_A_smithii with CRC-associated species in cases vs. controls. For each species, two stacked bars show the proportion of case (shown with circle) and control (shown with triangle) samples. Bars indicate four presence/absence patterns based on detection (non-zero relative abundance): both present (orange), only Methanobrevibacter_A_smithii (blue), only the CRC-associated bacterial species (green), and neither (grey). Numbers above the bars indicate the odds ratio (OR) and FDR-adjusted *p*-value (q) from Fisher's exact test calculated within each group of case and control. An OR > 1 denotes a tendency for co-occurrence (the other taxon is more likely when Methanobrevibacter_A smithii is present). Source data are provided as a Source Data file.

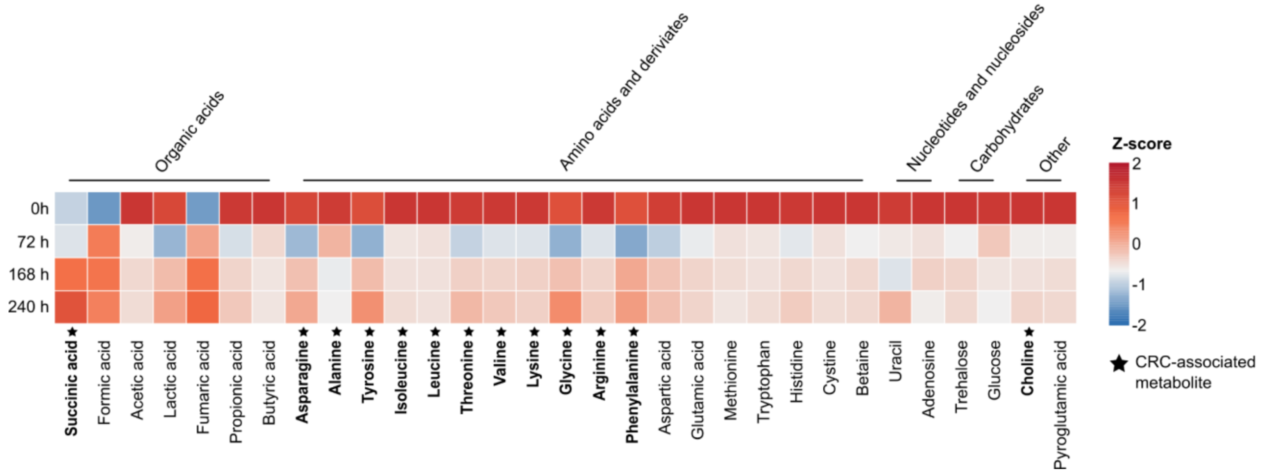

Supplementary Fig. 11. Uptake of amino acids and other compounds by *M. smithii* ALI. Metabolite analysis was performed using NMR spectroscopy on three biological replicates of *M. smithii* ALI, previously grown in MS medium and sampled at 0, 72, 168, and 240 h post-inoculation, as described in a previous study[5]. Each cell represents the Z-score of the respective metabolite, calculated across all samples as the number of standard deviations from the mean. Source data are provided as a Source Data file.

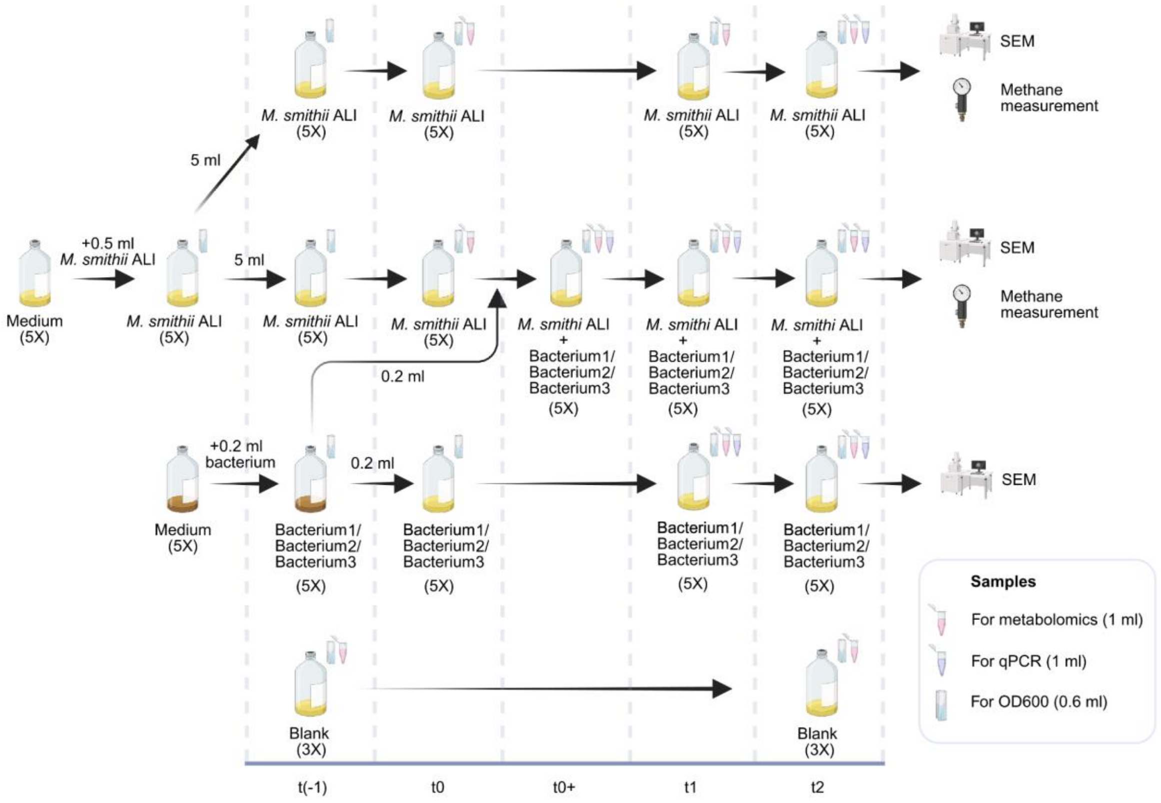

Supplementary Fig. 12. Experimental setup for co-culturing *Methanobrevibacter smithii* DSM 2375 (=ALI) with three CRC-associated bacterial strains, carried out separately: *Fusobacterium nucleatum* (DSM 15643) (=Bacterium 1), enterotoxigenic *Bacteroides fragilis* (DSM 2151) (=Bacterium 2), and *Escherichia coli* (=Bacterium 3) isolated from the fecal sample of a methane-producing subject previously shown to be co-occurring with *Methanobrevibacter smithii*[6]. Bacterial strains were first cultured overnight in BHI medium and subsequently inoculated into a combined BHI + MS medium under anaerobic conditions for co-cultivation. To account for differences in growth dynamics, 5 ml of *M. smithii* was pre-inoculated 24 h prior to bacterial addition, ensuring the archaeon had time to establish before faster-growing bacteria were introduced. Time points were defined relative to *M. smithii* ALI or bacterial inoculation: t(0) = 24 h post-inoculation, before the addition of bacteria, t(0+) = 24 h post inoculation of *M. smithii* ALI and right after the inoculation of bacterium; t(1) = 48 h post-archaeal inoculation, 24 h post-bacterial inoculation; t(2) = 96 h post-archaeal inoculation, 72 h post-bacterial inoculation. Samples were collected at key intervals for OD measurements, qPCR, metabolomics, and endpoint analyses including methane production, F420 fluorescence, and SEM. Experiments included five biological replicates for cultures and three replicates of medium control. The figure was created in BioRender (Neumann, C. (2026) https://BioRender.com/uyg8vy4).

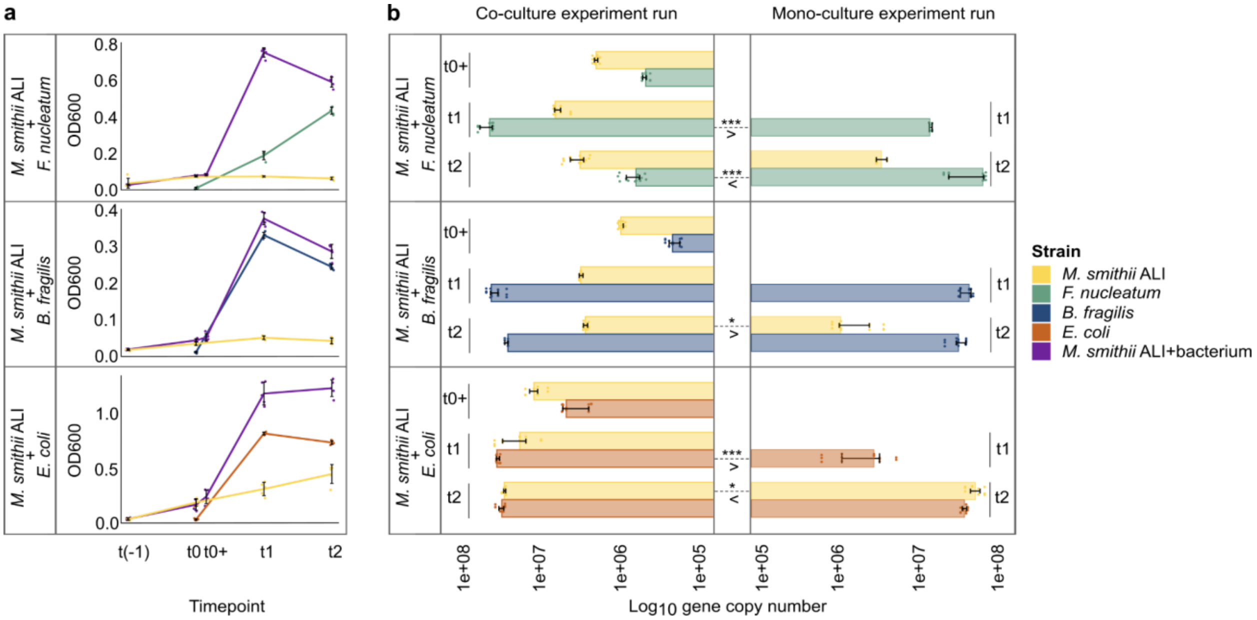

Supplementary Fig. 13. Growth dynamics and quantification of *Methanobrevibacter smithii* ALI and CRC-associated bacteria in mono- and co-culture setups. **a** Optical density (OD) measurements of *M. smithii* ALI, *Fusobacterium nucleatum*, *Bacteroides fragilis*, and *Escherichia coli* grown individually (n=5) and co-culture of these bacterial strains with *M. smithii* ALI (n=5). **b** Quantification of *M. smithii* ALI using *mcrA* gene copy number and of CRC-associated bacterial species (*F. nucleatum*, *B. fragilis*, and *E. coli*) using 16S rRNA gene copy number in mono- and co-culture conditions (n=3 technical replicates). Bacterial 16S rRNA gene copy numbers were normalized using species-specific values from the rrnDB database (*F. nucleatum* = 5 copies, *B. fragilis* = 6 copies, and *E. coli* = 7 copies). For each co-culture setup, mono-cultures of *M. smithii* ALI and the respective bacterial strain were grown in parallel under identical conditions. Time points were defined as t1: 24 h post bacterial inoculation (48 h post *M. smithii* ALI inoculation), and t2: 72 h post bacterial inoculation (96 h post *M. smithii* ALI inoculation). Pairwise differences were assessed using two-sided unpaired Wilcoxon rank-sum tests, with Benjamini–Hochberg FDR correction across comparisons. *$p < 0.05$; ***$p < 0.001$. *M. smithii ALI* + *F. nucleatum* (t1): *F. nucleatum* (t1): *p*-adjusted=5.31E-06; *M. smithii ALI* + *F. nucleatum* (t2): *F. nucleatum* (t2): *p*-adjusted=5.31E-06; *M. smithii ALI* + *B. fragilis* (t2): *M. smithii ALI* (t2): *p*-adjusted=0.0461 ; *M. smithii ALI* + *E. coli* (t1): *E. coli* (t1): *p*-adjusted=1.84E-05 ; *M. smithii ALI* + *E. coli* (2: *M. smithii ALI* (t2): *p*-adjusted=0.0327. Each dot represents a single biological replicate and error bars indicate standard deviations. Source data are provided as a Source Data file.

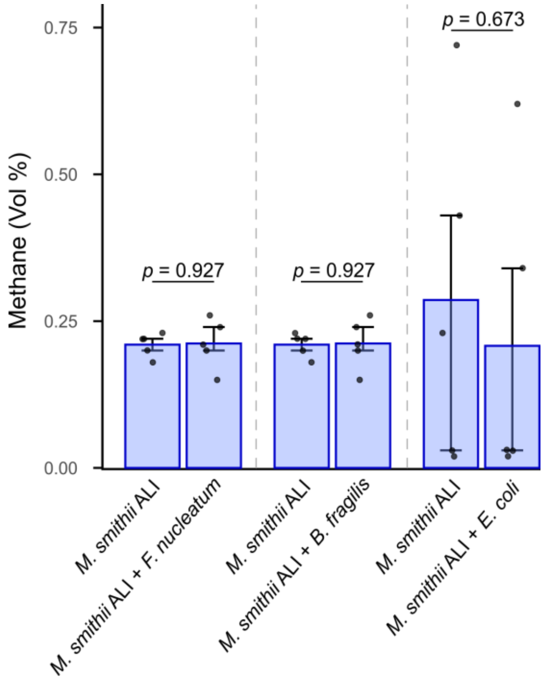

Supplementary Fig. 14. Methane concentrations produced by *Methanobrevibacter smithii* ALI are shown for both mono-culture and co-culture conditions with the respective bacterial strain (*Fusobacterium nucleatum*, *Bacteroides fragilis,* and *Escherichia coli*). Data points represent measurements from five biological replicates, and error bars indicate standard deviations. For each co-culture experiment, mono-cultures of *M. smithii* ALI and the corresponding bacterial strain were cultivated in parallel under identical conditions to allow direct comparison of methane production dynamics. Two-sided independent-samples Student's t-tests were used when both groups met normality assumptions (Shapiro–Wilk test, $p > 0.05$); otherwise, two-sided Wilcoxon rank-sum tests were applied. Source data are provided as a Source Data file.

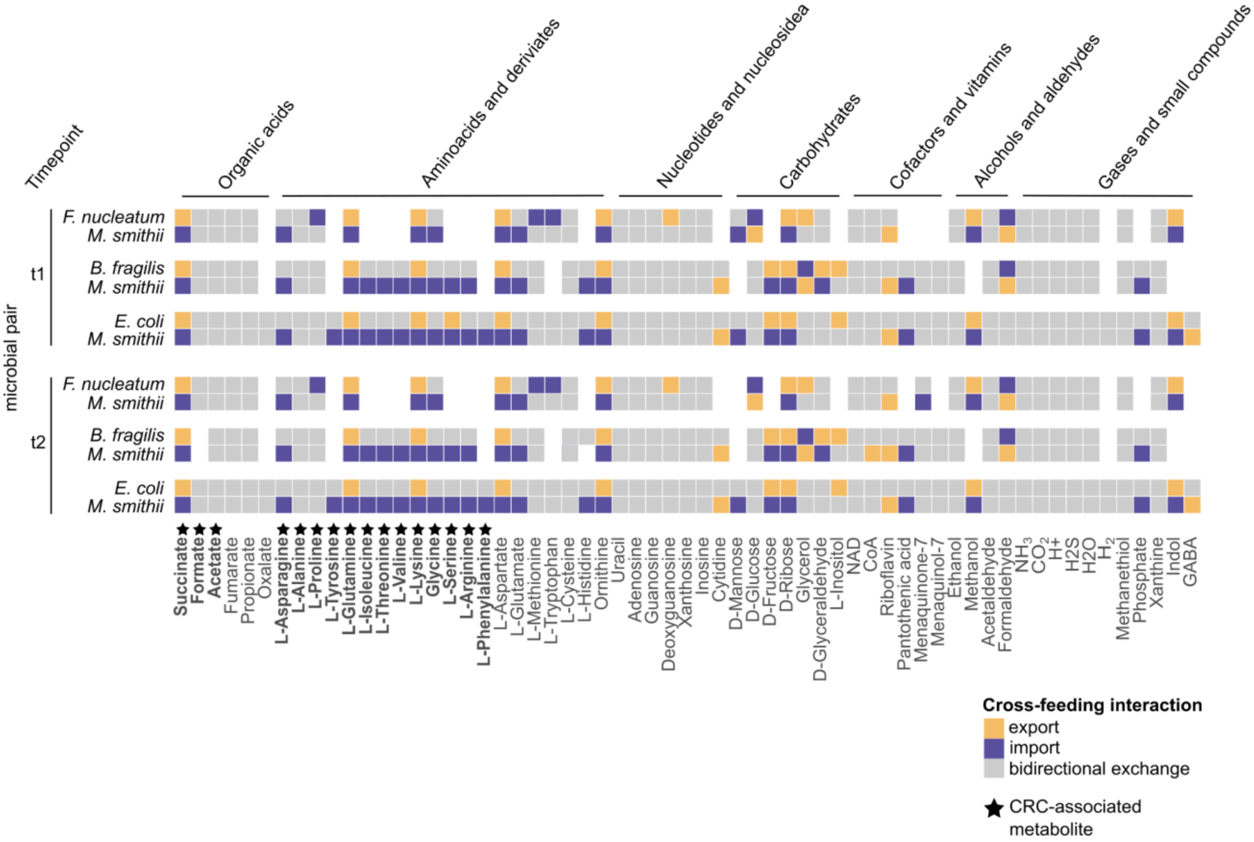

Supplementary Fig. 15. Predicted metabolite exchanges were based on relative abundances of each archaeon–bacterium pair in co-culture at two time points (t1 and t2), as determined by qPCR. At t1, mean relative abundances were: *Fusobacterium nucleatum* (=0.8845) and *Methanobrevibacter smithii* ALI (=0.1155); *Bacteroides fragilis* (=0.921) and *M. smithii* ALI (=0.0780); *Escherichia coli coli* (=0.6262) and *M. smithii* ALI (=0.3738). At t2: *F. nucleatum* (=0.9274) and *M. smithii* ALI (=0.0726); *B. fragilis* (=0.9840) and *M. smithii* ALI (=0.0160); *E. coli* (=0.5696) and *M. smithii* ALI (=0.4304) (Supplementary Data 7). Arrows represent predicted exchange of cross-fed metabolites, with color indicating directionality: export (yellow), import (purple), and bidirectional exchange (grey). Only metabolites exchanged between species are shown. Metabolites highlighted in bold have previously been implicated in CRC according to the literature.

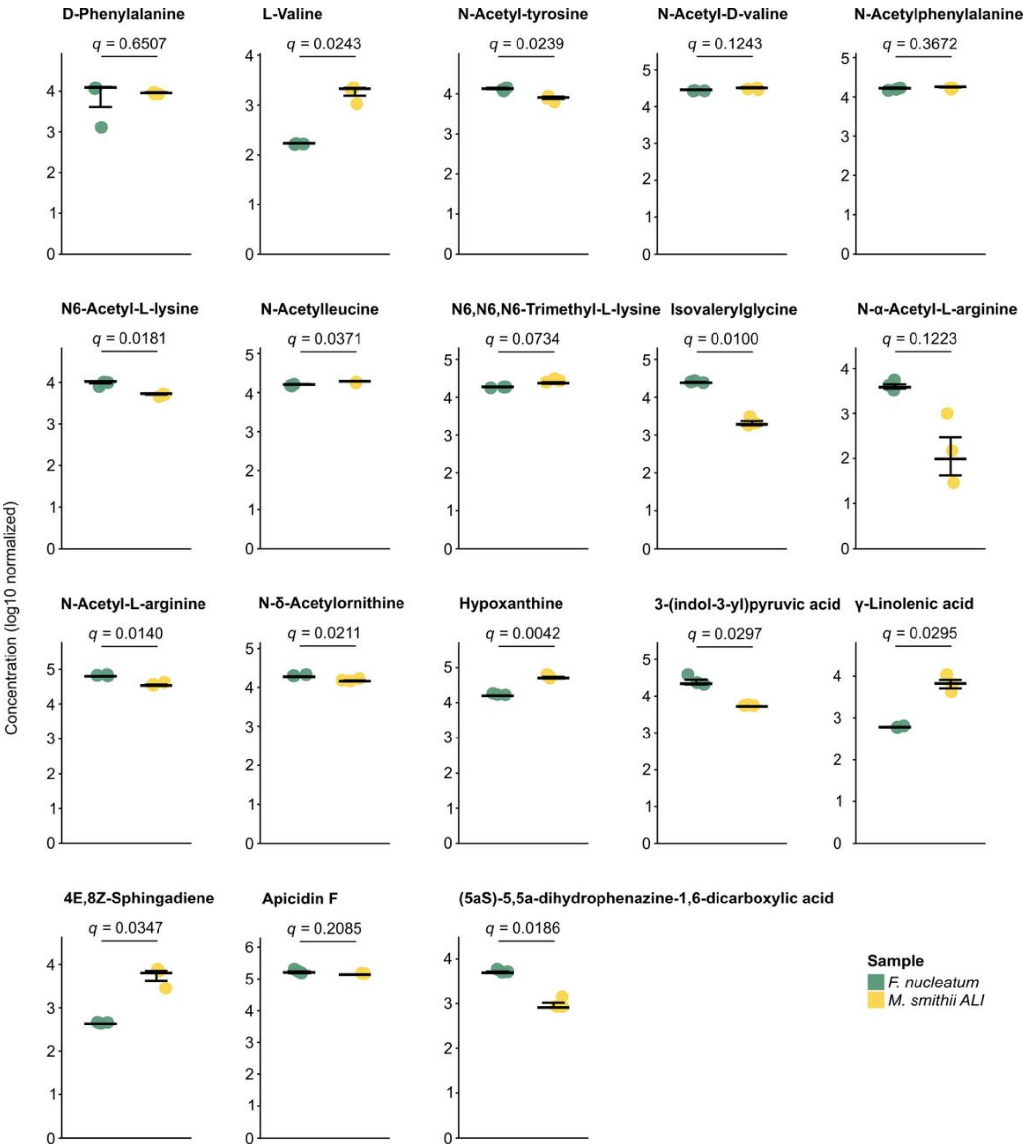

Supplementary Fig. 16. Scatter plot of metabolites with reported CRC-related effects (promoting or suppressive) based on literature. Metabolites were detected in the supernatants of optimally grown mono-cultures of *Fusobacterium nucleatum* (BHI medium) and *Methanobrevibacter smithii* ALI (BHI + MS medium) (n=3). Metabolite concentrations were normalized using PQN and $\log_{10}$-transformed to adjust for cell count differences and dilution effects. Statistical significance was assessed using a two-sided t-test, and *q*-values represent the FDR-adjusted *p*-values. Each dot represents a single biological replicate and error bars indicate standard deviations. Source data are provided as a Source Data file.

References

1. Yachida, S., *et al.* Metagenomic and metabolomic analyses reveal distinct stage-specific phenotypes of the gut microbiota in colorectal cancer. *Nature Medicine* **25**, 968-976 (2019).
2. Zeller, G., *et al.* Potential of fecal microbiota for early-stage detection of colorectal cancer. *Molecular Systems Biology* **10** (2014).
3. Vogtmann, E., *et al.* Colorectal cancer and the human gut microbiome: reproducibility with whole-genome shotgun sequencing. *PLOS ONE* **11**, e0155362 (2016).
4. Hannigan, G.D., Duhaime, M.B., Ruffin, M.T., Koumpouras, C.C. & Schloss, P.D. Diagnostic potential and interactive dynamics of the colorectal cancer virome. *mBio* **9**(2018).
5. Weinberger, V., *et al.* Expanding the cultivable human archaeome: Methanobrevibacter intestini sp. nov. and strain Methanobrevibacter smithii ‘GRAZ-2’ from human faeces. *International Journal of Systematic and Evolutionary Microbiology* **75** (2025).
6. Duller, S., *et al.* Targeted isolation of Methanobrevibacter strains from fecal samples expands the cultivated human archaeome. *Nature Communications* **15**, 7593 (2024).
