# Supplementary material for: Cross-domain metabolic interactions link Methanobrevibacter smithii to colorectal cancer microbial ecosystems
Source: Nat Commun. 2026 Feb 20;17:2979. doi: 10.1038/s41467-026-69711-7 (PMC13035836; doi:10.1038/s41467-026-69711-7)
Supplement: Supplementary file 2 — Description of Additional Supplementary Files [file 41467_2026_69711_MOESM2_ESM.pdf]

Description of Additional Supplementary Files

Manuscript: **Cross-domain metabolic interactions link *Methanobrevibacter smithii* to colorectal cancer microbial ecosystems**

Associated supporting files referenced in the Supplementary Information are inventoried below.

Item	Type	Brief description
Supplementary Data 1	Excel sheet	Details of the studies and fecal metagenome samples included in the meta-analysis
Supplementary Data 2	Excel sheet	Differential abundance analysis of the five most prevalent archaeal species
Supplementary Data 3	Excel sheet	Differential abundance analysis of other archaeal species
Supplementary Data 4	Excel sheet	Literature review for the curation of a set of twelve bacterial taxa identified as CRC microbial biomarkers
Supplementary Data 5	Excel sheet	Differential abundance analysis of the CRC-associated bacterial taxa
Supplementary Data 6	Excel sheet	Correlation analysis between these CRC-associated bacterial taxa and Methanobrevibacter_A_smithii
Supplementary Data 7	Excel sheet	Metabolomics of *M. smithii* ALI at different timepoints
Supplementary Data 8	Excel sheet	qPCR results of bacterial and archaeal monocultures/cocultures

Supplementary Data 9	Excel sheet	Metabolomics (NMR) profile of *F. nucleatum*, *B. fragilis*, *E. coli* and *M. smithii* in mono culture and coculture (data presented as integrals)
Supplementary Data 10	Excel sheet	Metabolomics (NMR) profile of *F. nucleatum*, *B. fragilis*, *E. coli* and *M. smithii* ALI in mono culture and coculture shown for metabolites significantly higher in co-culture compared to moncultures (data presented as concentrations)
Supplementary Data 11	Excel sheet	Results of the Welch's t-test and the Wilcoxon rank-sum test used to compare co-cultures and mono- cultures across individual NMR metabolomic profiles
Supplementary Data 12	Excel sheet	Mass-spec profile of *F. nucleatum* + *M. smithii* ALI co-culture supernatant as well as blank medium (triplicates)
Supplementary Data 13	Excel sheet	Comparison of Mass-spec profile between *F. nucleatum* and *M. smithii* in monocultures (triplicates)
