# Supplementary material for: Cross-domain metabolic interactions link Methanobrevibacter smithii to colorectal cancer microbial ecosystems
Source: Nat Commun. 2026 Feb 20;17:2979. doi: 10.1038/s41467-026-69711-7 (PMC13035836; doi:10.1038/s41467-026-69711-7)
Supplement: Supplementary file 16 — Reporting Summary [file 41467_2026_69711_MOESM16_ESM.pdf]

nature portfolio

Corresponding author(s): Christine Moissl-Eichinger

Last updated by author(s): Jan 30, 2026

Reporting Summary

Nature Portfolio wishes to improve the reproducibility of the work that we publish. This form provides structure for consistency and transparency in reporting. For further information on Nature Portfolio policies, see our Editorial Policies and the Editorial Policy Checklist.

Statistics

For all statistical analyses, confirm that the following items are present in the figure legend, table legend, main text, or Methods section.

n/a	Confirmed	
☐	☒	The exact sample size (*n*) for each experimental group/condition, given as a discrete number and unit of measurement
☐	☒	A statement on whether measurements were taken from distinct samples or whether the same sample was measured repeatedly
☐	☒	The statistical test(s) used AND whether they are one- or two-sided<br>*Only common tests should be described solely by name; describe more complex techniques in the Methods section.*
☐	☒	A description of all covariates tested
☐	☒	A description of any assumptions or corrections, such as tests of normality and adjustment for multiple comparisons
☐	☒	A full description of the statistical parameters including central tendency (e.g. means) or other basic estimates (e.g. regression coefficient) AND variation (e.g. standard deviation) or associated estimates of uncertainty (e.g. confidence intervals)
☐	☒	For null hypothesis testing, the test statistic (e.g. *F*, *t*, *r*) with confidence intervals, effect sizes, degrees of freedom and *P* value noted<br>*Give P values as exact values whenever suitable.*
☒	☐	For Bayesian analysis, information on the choice of priors and Markov chain Monte Carlo settings
☒	☐	For hierarchical and complex designs, identification of the appropriate level for tests and full reporting of outcomes
☒	☐	Estimates of effect sizes (e.g. Cohen's *d*, Pearson's *r*), indicating how they were calculated

Our web collection on statistics for biologists contains articles on many of the points above.

Software and code

Policy information about availability of computer code

Data collection	not applicable
Data analysis	standard scripts (no code designed) are uploaded to Github repository https://github.com/CME-lab-research/archaeome-disease-profiling/. The following software and tools where used: SRA Toolkit v3.1.0, BBMap suite v39.01 (removehuman.sh, repair.sh), GRCh38 human reference genome, fastp v0.23.4, Kraken2 v2.1.2, Bracken v2.7, Unified Human Gastrointestinal Genome (UHGG) database v2.0.1, Kraken2 Standard Database, R v4.3.1, RStudio v2023.06.1+524, MMUPHin R package v1.23, phyloseq R package v1.44.0, vegan R package, Python v3.10, scikit-learn v1.5, shap v0.44, gapseq v1.4.0 (development version, commit acb9647), PyCoMo v0.2.7, ScyNet, Cytoscape v3.10.0, GTDB release r226, rrnDB, Bio-Rad CFX Manager Software v3.1, TopSpin v4.5, MATLAB v2014b, timsControl® software, Compass HyStar® software, MetaboScape® v2024b, Human Metabolome Database (HMDB), NIST Mass Spectral Library, METLIN database, MiMeDB database, Pacific Northwest National Laboratory (PNNL) CCS database, MetaboAnalyst v6.0.

For manuscripts utilizing custom algorithms or software that are central to the research but not yet described in published literature, software must be made available to editors and reviewers. We strongly encourage code deposition in a community repository (e.g. GitHub). See the Nature Portfolio guidelines for submitting code & software for further information.

Data

Policy information about availability of data

All manuscripts must include a data availability statement. This statement should provide the following information, where applicable:

- Accession codes, unique identifiers, or web links for publicly available datasets
- A description of any restrictions on data availability
- For clinical datasets or third party data, please ensure that the statement adheres to our policy

data availability statement is ialso ncluded in the manuscript. Raw sequencing data for stool samples All sequencing data analyzed in this study were obtained from previously published datasets and are publicly available from the European Nucleotide Archive (ENA) and the NCBI BioProject/SRA databases under the following accession numbers: PRJDB4176 [https://www.ebi.ac.uk/ena/browser/view/PRJDB4176], PRJEB6070 [https://www.ebi.ac.uk/ena/browser/view/PRJEB6070], PRJEB7774 [https://www.ebi.ac.uk/ena/browser/view/PRJEB7774], PRJEB10878 [https://www.ebi.ac.uk/ena/browser/view/PRJEB10878], PRJNA389927 [https://www.ncbi.nlm.nih.gov/bioproject/PRJNA389927], PRJEB12449 [https://www.ebi.ac.uk/ena/browser/view/PRJEB12449], PRJEB27928 [https://www.ebi.ac.uk/ena/browser/view/PRJEB27928], PRJNA447983 [https://www.ncbi.nlm.nih.gov/bioproject/PRJNA447983], PRJNA531273 [https://www.ncbi.nlm.nih.gov/bioproject/PRJNA531273] and PRJNA397112 [https://www.ncbi.nlm.nih.gov/bioproject/PRJNA397112], PRJNA400072 [https://www.ncbi.nlm.nih.gov/bioproject/PRJNA400072], SRA045646 [https://www.ncbi.nlm.nih.gov/sra/SRA045646] and SRA050230 [https://www.ncbi.nlm.nih.gov/sra/SRA050230], PRJEB32762 [https://www.ebi.ac.uk/ena/browser/view/PRJEB32762], PRJEB47976 [https://www.ebi.ac.uk/ena/browser/view/PRJEB47976], PRJNA798058 [https://www.ncbi.nlm.nih.gov/bioproject/PRJNA798058], PRJEB29127 [https://www.ebi.ac.uk/ena/browser/view/PRJEB29127], PRJNA834801 [https://www.ncbi.nlm.nih.gov/bioproject/PRJNA834801], PRJNA743718 [https://www.ncbi.nlm.nih.gov/bioproject/PRJNA743718], PRJEB53401 [https://www.ebi.ac.uk/ena/browser/view/PRJEB53401], and PRJEB17784 [https://www.ebi.ac.uk/ena/browser/view/PRJEB17784]. The NMR raw data generated in this study are available in Zenodo at https://doi.org/10.5281/zenodo.16311518. the LC-MS raw data generated in this study are available in Zenodo at https://doi.org/10.5281/zenodo.16367666. All additional data generated and analyzed in this study including gapseq outputs (including genome-scale metabolic models, gap-filled models, reaction and gene annotations, as well as pathway and transporter predictions), PyCoMo outputs (including flux variability analyses, metabolite secretion and uptake predictions, and community are publicly available in our GitHub repository (https://github.com/CME-lab-research/archaeome-disease-profiling/).

Research involving human participants, their data, or biological material

Policy information about studies with human participants or human data. See also policy information about sex, gender (identity/presentation), and sexual orientation and race, ethnicity and racism.

Reporting on sex and gender	The sex of the subjects was determined based on publicly available metadata from previously published metagenomic datasets used in this study. selected data were matched accordingly, where applicable.
Reporting on race, ethnicity, or other socially relevant groupings	NA
Population characteristics	No new cohort was recruited in this study and population characteristics were determined based on publicly available metadata from previously published metagenomic datasets used in this study.
Recruitment	NA
Ethics oversight	NA

Note that full information on the approval of the study protocol must also be provided in the manuscript.

Field-specific reporting

Please select the one below that is the best fit for your research. If you are not sure, read the appropriate sections before making your selection.

☒ Life sciences ☐ Behavioural & social sciences ☐ Ecological, evolutionary & environmental sciences

For a reference copy of the document with all sections, see nature.com/documents/nr-reporting-summary-flat.pdf

Life sciences study design

All studies must disclose on these points even when the disclosure is negative.

Sample size	Sample sizes for analyses and displays are given in respective Tables, or Figure legends. Sample sizes were determined by dataset availability from public fecal shotgun metagenomic studies that met pre-specified inclusion criteria (2000–August 30, 2024; adult participants; ≥20 cases; availability of required metadata or case–control matching; paired-end sequencing). No prospective sample size calculation was performed for these analyses because the study represents a retrospective aggregation of existing datasets. Instead, minimum sample thresholds and stratification rules were applied to ensure statistical robustness, including exclusion of cohorts with fewer than 20 cases, avoidance of stage-stratified colorectal cancer analyses when post-matching stage strata contained fewer than 10 samples, exclusion of stage 0 groups with fewer than five samples, and collapsing colorectal cancer stages into I/II and III/IV to increase effective sample size per comparison.
Data exclusions	No data were excluded, all information is available in Supplementary Data.

Replication	biological (min 3) and technical replicates were performed. All information is provided in legends and tables.
Randomization	not applicable
Blinding	not applicable

Reporting for specific materials, systems and methods

We require information from authors about some types of materials, experimental systems and methods used in many studies. Here, indicate whether each material, system or method listed is relevant to your study. If you are not sure if a list item applies to your research, read the appropriate section before selecting a response.

Materials & experimental systems

n/a	Involved in the study
☒	☐ Antibodies
☒	☐ Eukaryotic cell lines
☒	☐ Palaeontology and archaeology
☒	☐ Animals and other organisms
☒	☐ Clinical data
☒	☐ Dual use research of concern
☒	☐ Plants

Methods

n/a	Involved in the study
☒	☐ ChIP-seq
☒	☐ Flow cytometry
☒	☐ MRI-based neuroimaging

Plants

Seed stocks	*Report on the source of all seed stocks or other plant material used. If applicable, state the seed stock centre and catalogue number. If plant specimens were collected from the field, describe the collection location, date and sampling procedures.*
Novel plant genotypes	*Describe the methods by which all novel plant genotypes were produced. This includes those generated by transgenic approaches, gene editing, chemical/radiation-based mutagenesis and hybridization. For transgenic lines, describe the transformation method, the number of independent lines analyzed and the generation upon which experiments were performed. For gene-edited lines, describe the editor used, the endogenous sequence targeted for editing, the targeting guide RNA sequence (if applicable) and how the editor was applied.*
Authentication	*Describe any authentication procedures for each seed stock used or novel genotype generated. Describe any experiments used to assess the effect of a mutation and, where applicable, how potential secondary effects (e.g. second site T-DNA insertions, mosiacism, off-target gene editing) were examined.*
